# Surface charge modulation of rifampicin-loaded PLA nanoparticles to improve antibiotic delivery in *Staphylococcus aureus* biofilms

**DOI:** 10.1186/s12951-020-00760-w

**Published:** 2021-01-07

**Authors:** David Da Costa, Chloé Exbrayat-Héritier, Basile Rambaud, Simon Megy, Raphaël Terreux, Bernard Verrier, Charlotte Primard

**Affiliations:** 1 Laboratoire de Biologie Tissulaire Et Ingénierie Thérapeutique, UMR5305, CNRS/Université, Lyon 1, Lyon, France; 2 Adjuvatis, 7 passage du Vercors, 69007 Lyon, France

**Keywords:** Biofilm, PLA nanoparticles, Antibiotic, Delivery, Surface charge, Interaction

## Abstract

**Background:**

After the golden age of antibiotic discovery, bacterial infections still represent a major challenge for public health worldwide. The biofilm mode of growth is mostly responsible for chronic infections that current therapeutics fail to cure and it is well-established that novel strategies must be investigated. Particulate drug delivery systems are considered as a promising strategy to face issues related to antibiotic treatments in a biofilm context. Particularly, poly-lactic acid (PLA) nanoparticles present a great interest due to their ability to migrate into biofilms thanks to their submicronic size. However, questions still remain unresolved about their mode of action in biofilms depending on their surface properties. In the current study, we have investigated the impact of their surface charge, firstly on their behavior within a bacterial biofilm, and secondly on the antibiotic delivery and the treatment efficacy.

**Results:**

Rifampicin-loaded PLA nanoparticles were synthetized by nanoprecipitation and characterized. A high and superficial loading of rifampicin, confirmed by an in silico simulation, enabled to deliver effective antibiotic doses with a two-phase release, appropriate for biofilm-associated treatments. These nanoparticles were functionalized with poly-l-lysine, a cationic peptide, by surface coating inducing charge reversal without altering the other physicochemical properties of these particles. Positively charged nanoparticles were able to interact stronger than negative ones with *Staphylococcus aureus*, under planktonic and biofilm modes of growth, leading to a slowed particle migration in the biofilm thickness and to an improved retention of these cationic particles in biofilms. While rifampicin was totally ineffective in biofilms after washing, the increased retention capacity of poly-l-lysine-coated rifampicin-loaded PLA nanoparticles has been associated with a better antibiotic efficacy than uncoated negatively charged ones.

**Conclusions:**

Correlating the carrier retention capacity in biofilms with the treatment efficacy, positively charged rifampicin-loaded PLA nanoparticles are therefore proposed as an adapted and promising approach to improve antibiotic delivery in *S. aureus* biofilms.

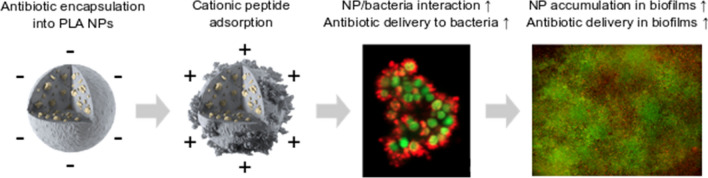

## Introduction

Despite a relentless fight against infectious agents that have caused high mortality and morbidity rates all around the world for centuries, the perpetual emergence of new microbes makes infectious diseases a major public health burden representing constant challenges for human survival and life quality [1]. Among these diseases, it is estimated that about 80% of bacterial infections are associated with biofilms [2]. Biofilms are defined as sessile and organized communities of single or multi-species bacteria adhering to biotic or abiotic surfaces and living within an auto-produced matrix of extracellular polymeric substances composed of polysaccharides, proteins, nucleic acids and lipids [3]. Such lifestyle allows bacteria to escape the immune system and may require up to 1,000 times the minimal inhibitory concentration of antibiotics for an effective treatment, leading to therapeutic failures [4]. This biofilm recalcitrance involves two phenomena. On the one hand, acquired by horizontal gene transfer and mutagenesis, antibio-resistance is facilitated under biofilm conditions due to high genetic diversity and sub-inhibitory concentrations of antibiotics exposed to bacteria [5]. On the other hand, tolerance toward antibiotics is multifactorial: (1) the biofilm matrix acts as a barrier against antibiotics due to its physicochemical properties, (2) the microenvironment with gradients of pH, oxygen and nutrients can alter antibiotic effects and (3) the presence of persister bacteria which exist in a dormant state and are insensitive to antibiotic treatments. Antibiotic delivery in biofilms is then a challenging task in the fight against biofilms [6].

Nanotechnology for antimicrobial drug delivery has been widely explored over the last few decades [7–9]. Particularly, poly-lactic acid (PLA) and poly-lactic-co-glycolic acid (PLGA) biodegradable nanoparticles (NPs) have attracted a great interest in this field [10]. They can be synthesized by various processes and they result in solid colloidal particles ranging from nanometers to micrometers, allowing the encapsulation or surface adsorption of a wide range of bioactive molecules, either lipophilic or hydrophilic [11]. They present several advantages thanks to their properties, like biocompatibility, biodegradability and versatility of functionalization [12]. Also, PLA and PLGA NPs display a high structural integrity leading to highly stable drug delivery systems [13, 14]. Their capacities to improve drug pharmacokinetics and bioavailability, by protecting drug against inactivation by enzymes, by targeting and penetrating biofilms or by favoring a sustained drug release while limiting side effects, make these systems an adapted strategy to treat biofilm infections [6].

While most of the studies focused on the ability of these systems to eradicate biofilms, little is known about their mode of action in biofilms [15]. It is yet important to evaluate this fundamental aspect to improve the design and the functionality of these carriers, to efficiently deliver antibiotics in biofilms. Indeed, NP physicochemical properties play a crucial role in the sequential steps responsible for their interaction with biofilms: (1) the transport of NPs to the biofilms, (2) their attachment to the biofilm surface, (3) their migration within the matrix and (4) their accumulation [15, 16]. Physicochemical properties of NPs as drug carriers are mainly determined by their size and their surface properties, especially the surface charge (anionic, neutral or cationic). In term of NP size, Liu et al*.* defined that submicron NPs were optimal compared to microparticles, better penetrating biofilms considering the size of biofilm water channels, and impeding the recognition by the immune system and body clearance [17]. The penetration of NPs has been studied in more details into *Burkholderia multivorans* and *Pseudomonas aeruginosa* biofilms, in which the size cutoff of NPs able to penetrate into dense biofilm clusters in larger quantities was around 130 nm among tested diameters ranging from 40 to 550 nm [18].

Regarding the NP surface charge, it can be responsible for non-specific biofilm targeting by electrostatic interactions with both bacteria and the matrix, favoring the attachment of NPs to biofilms [6, 15]. Forier et al*.* highlighted that carboxylate-modified polystyrene NPs (anionic) of 220 nm in size were able to migrate in biofilms while dimethylamine-ethylamine-modified NPs (cationic) stuck at the entry of bacteria-packed clusters, hypothesizing that interactions with anionic macromolecules of the matrix, like extracellular DNA, inhibited cationic NP penetration [19, 20]. Likewise, poly(β-amino ester)-modified micelles of 80 nm and poly(amino propyl imidazole-aspartate)-modified silver nanoclusters of 8 nm have been shown to accumulate in biofilms thanks to increased interactions between those positively charged systems and negatively charged bacteria [21, 22]. While negatively charged carriers were washed away from biofilms, cationic ones prevented their wash out by water. The impact of such antibiotic-loaded NP accumulation in the vicinity of bacteria on their capacity to sustainably deliver effective antibiotic concentrations after washing was demonstrated by Hasan et al*.* on planktonic bacteria [23]. After a short contact time, washing fully removed free antibiotic and negatively charged NPs non-interacting with bacteria, leading to bacteria survival. On the contrary, positively charged polyethylenimine-coated NPs, which were shown to adhere to planktonic bacteria, were able to provide antiplanktonic activity by locally and sustainably delivering the encapsulated antibiotic to bacteria after washing. The ability of antibiotic-loaded NPs to passively target biofilms by electrostatic interactions may thus represent a predominant point for antibiotic activity. However, this effect remains poorly described in biofilms, despite a great interest [6, 15].

The aim of this prospective study was to evaluate the impact of surface charge of submicronic PLA NPs on the antibiotic delivery in biofilms, through (1) the ability of negatively and positively charged NPs to interact with planktonic bacteria, (2) their capability to migrate and to accumulate into biofilms, and (3) the effect of this passive targeting and accumulation on the efficacy of antibiotic treatment on planktonic and biofilm modes of growth respectively. *Staphylococcus aureus* was chosen as the biofilm model because of its clinical relevance, where it is involved in various chronic and severe diseases like osteomyelitis, endocarditis, urinary tract infections, implant-associated infections or wound infections [24]. PLA NPs were loaded with rifampicin (RIF), a broad-spectrum antibiotic including *S. aureus*, and surface functionalized by poly-l-lysine (PLL), a cationic peptide, to reverse negative NP surface charge to positive.

## Material and methods

### Preparation of drug-loaded and charge-reversed PLA NPs

Poly(D,L-lactic acid) (PLA, MW = 45–80 kDa, Merck, Switzerland) NPs were synthetized by a surfactant-free process of nanoprecipitation adapted from Fessi et al*.* [25]. Briefly, the polymer was dissolved at 2% (w/v) in acetone (Carlo Erba, France). This organic phase was flowed by a fast drop-by-drop into an aqueous phase composed of 2 mM sodium bicarbonate (Fisher Scientific, France) and ethanol (Carlo Erba, France) in a 6:4 (v/v) proportion under moderate stirring. Both organic solvents were then evaporated under low pressure by a rotary evaporator (Rotavapor R210, Buchi, France) at 30 °C. Final NP suspensions were stored at 4 °C.

For their loading into NPs, rifampicin (RIF, MW = 823 Da, Euromedex, France) or a fluorophore (Dy650-decylamide, Dyomics, Germany) were added to the organic phase before processing nanoprecipitation. α-poly-l-lysine (PLL, MW = 16 kDa, Alamanda Polymers, USA) was adsorbed on the NP surface by adding 1000 µg/mL volume to volume in the NP suspension (0.5% of solid content) under slow rotary stirring for 1 h at room temperature.

### Nanoparticle characterization

The solid content of NPs was defined as the proportion (w/v) of PLA polymer into the suspension and was measured by weighing the NP suspension before and after drying.

NP hydrodynamic diameter and size distribution defined by the polydispersity index (PDI) were evaluated by dynamic light scaterring (DLS) using a Zetasizer Nano ZS (Malvern Panalytical, UK) by diluting NP suspensions into a 0.22 µm-filtered 1 mM NaCl solution. Zeta potential was calculated after measurement of the NP electrophoretic mobility using the same apparatus Zetasizer Nano ZS.

NPs were also characterized by morphological observations with scanning electron microscopy (SEM). Briefly, a drop of NPs diluted in water to 0.1% of solid content was deposited on glass coverslips and stuck on metal stubs, before air drying under low pressure. The dried stubs were then sputtered by 10 nm of copper with a metallizer (BAL-TEC MED020, Leica Microsystems, France) before SEM observations (MERLIN VP Compact, Zeiss, CTµ, Centre Technologique des Microstructures, Lyon 1, France) with an acceleration voltage of 10 kV.

### Drug loading

Charge-reversed and antibiotic-loaded NPs (NP-RIF and NP-RIF-PLL) were centrifuged 20 min at 16,000×*g*. The supernatant containing the non-loaded RIF fraction was separated from the pellet, which consisted of the NP-loaded RIF. Both samples were vacuum dried for 2 h at 25 °C before resuspension with acetonitrile (Carlo Erba, France) in order to fully dissolve PLA and RIF. Solutions were quantified by microplate spectrophotometry (Multiskan GO, Thermo Scientific, France) measuring RIF absorbance at 480 nm and correlating with a linear calibration curve of RIF into acetonitrile (R^2^ = 0.99996), ranging from 8 to 256 µg/mL. The loading efficiency and the loading rate were calculated by the following equations.

$$Loading efficiency=100\times \frac{Mass of loaded RIF into NPs}{Total mass of RIF}$$

$$Loading rate=100\times \frac{Mass of loaded RIF into NPs}{Mass of PLA}$$

Charge-reversed and fluorophore-loaded NPs (NP-Dy650 and NP-Dy650-PLL) were centrifuged 20 min at 16,000×*g*. The supernatant containing the non-loaded Dy650 fraction and the total NP suspension containing all the Dy650 were directly quantified by microplate spectrofluorimetry (Infinite M1000, Tecan, Switzerland). Fluorescence was measured by excitation at 655 nm and emission at 675 nm. The loaded Dy650 proportion into NPs was calculated by subtracting the non-loaded Dy650 fraction from the total proportion. The loading efficiency and the loading rate were then calculated as described above.

### Cell toxicity evaluation

Murine macrophages (Raw 264.7, ATCC, USA) and fibroblasts (NIH/3T3, ATCC, USA) were cultured into Dulbecco's modified eagle medium supplemented with 10% of fetal bovine serum and 1% of penicillin/streptomycin (DMEM, Gibco, Fisher Scientific, France) at 37 °C in a humidified 5% CO_2_ atmosphere. Briefly, 5 × 10^4^ cells per well (200 µL) were seeded in a 96-well microplate (Corning, Fisher Scientific, France) and the plate was incubated for 24 h. Then, 50 µL of four-fold serial dilutions of charge-reversed, antibiotic-loaded and plain NPs, ranging from 0.008 to 8 mg/mL of NP concentration, were added and incubated for another 24 h at 37 °C and 5% CO_2_. Controls containing only cells (untreated) or medium without cells (blank) were also included. Cell viability was evaluated by adding 25 µL of PrestoBlue reagent (Invitrogen, Fisher Scientific, France) in wells for an additional 2 h. Fluorescence was measured by excitation at 535 nm and emission at 615 nm using a microplate reader (Tecan, Switzerland). Cell viability was assessed as a percentage related to the untreated control.

### In silico simulations

Dissipative Particle Dynamics (DPD) simulations were performed using the Material Studio software (Biovia, United States). For the water molecules (W), a coarse graining approach was used in which one bead represents 3 molecules of water. The radius of the water bead was set to 3.23 Å and its molecular mass to 54 Da. RIF molecules were constructed using 3 different types of beads: R1, R2 and R3. PLA molecules were constructed as linear repetitions of 70 units of lactic acid monomers (LA)_n_ and PLL molecules were constructed as linear repetitions of 10 units of lysine monomers (K)_n_. The lysine molecule itself was constructed using 2 different beads, one for its backbone and one for its side chain. For the calculations, all the solubility parameters δ_i_ were calculated with the Material Studio software using either the Synthia module for polymers, or models constructed with the Amorphous Cell module, or a combination of both methods. For the models constructed as amorphous cells, all the calculations were performed with the Forcite module using the COMPASS II force field for atom parameters and partial charges. These solubility parameters were then used to calculate the Flory–Huggins interaction parameters χ_ij_ for the corresponding binary mixtures, which were calculated using the relation χ_ij_ = (v/RT)(δ_i_—δ_j_)^2^ where R is the gas constant, T the absolute temperature and v the mean volume per mole of the two corresponding components [26]. The molar volume for each component was determined using the MOE software. The Flory–Huggins interaction parameters were then converted into DPD repulsion parameters a_ij_, which were obtained using the relation a_ij_ = 25 + 3.50 χ_ij_ [27]. Our calculations were performed using 280^3^ Å cubic periodic boxes of water as starting points for all the dynamics. Droplets with a radius of 130 Å containing a mixture of water, PLA and RIF molecules were placed at their center. In order to mimic the experimental ratio of PLA and RIF molecules during the NP synthesis, we used a mixture ratio of 137 water molecules versus 81 PLA molecules and 10 RIF molecules, leading to a loading rate of 2%. For the simulations involving PLL chains with a PLL/PLA mass ratio of 1%, the number of RIF molecules was set up at 316 PLL chains for 8100 PLA molecules. DPD experiments were conducted using a time scale of 3 ps and various total simulation times, up to 30 ns.

### In vitro drug release

Charge-reversed and antibiotic-loaded NPs were diluted in 6 mL of phosphate buffer saline (PBS, Gibco, Fisher Scientific, France) and stored at 37 °C under mild stirring for 48 h. At predetermined times, 300 µL of NP suspension were removed and replaced with fresh buffer to keep the volume constant. The withdrawn samples were centrifuged 20 min at 16,000×*g* and rifampicin in the pellets and supernatants was quantified, as described above for drug loading. Fresh RIF-loaded NPs were stored at 4 °C for 60 days to evaluate their drug loading as described above.

### Bacterial strain and growth conditions

*Staphylococcus aureus* SH1000 strain was kindly provided by the team Staphylococcal pathogenesis, International Center for Infectiology Research, France. For each experiment, a defrost glycerol stock of *S. aureus* was plated on brain heart infusion (BHI) agar medium (Sigma-Aldrich, France) overnight at 37 °C. For planktonic bacteria assays, some colonies were inoculated into BHI liquid medium (Becton Dickinson, France) for 24 h at 37 °C. For biofilm assays, BHI was supplemented with 1% glucose (Sigma-Aldrich, France). Bacterial growth was then measured by cuvette spectrophotometry (BioPhotometer, Eppendorf, France) at 600 nm.

### Antibiotic susceptibility test on planktonic bacteria

The minimum inhibitory concentration (MIC) on planktonic *S. aureus* was determined by using the broth microdilution method adapted from Wiegand et al*.* [28]. Briefly, an overnight culture of *S. aureus* was diluted into BHI to an optical density (OD) of 0.008 at 600 nm corresponding to 1 × 10^6^ CFU/mL and 100 µL of this suspension were dispensed into a non-treated, tissue culture microplate (Corning, Fisher Scientific, France). The same volume of four-fold serial dilutions of RIF or equivalent concentrations into NP suspensions without or with PLL coating (NP-RIF and NP-RIF-PLL), ranging from 0.06 ng/mL to 64 µg/mL was added. PLL, plain NPs and plain NP-PLL were also tested in equivalent concentrations to NP-RIF and NP-RIF-PLL. Controls containing only bacteria (untreated) or medium without cells (blank) were also tested. The microplate was then incubated at 37 °C aerobically for 24 h and the absorbance at 600 nm was read using a microplate spectrophotometer (Multiskan GO, Thermo Scientific, France). The MIC was defined as the lowest drug concentration that prevents planktonic bacterial growth.

### In vitro antibiofilm tests on 24 h-old biofilms

Antibiofilm activity of RIF, NP-RIF and NP-RIF-PLL was investigated on *S. aureus* biofilms by crystal violet (CV, Sigma-Aldrich, France) staining. In brief, an overnight culture of *S. aureus* suspension was diluted into BHI supplemented with 1% glucose to an OD_600_ of 0.004 corresponding to 5 × 10^5^ CFU/mL. Bacteria were seeded into a non-treated, tissue culture 96-well microplate with 100 µL per well and incubated at 37 °C aerobically for 24 h. After incubation, formed biofilms were washed three times with PBS. The same volume of four-fold serial dilutions of RIF or equivalent concentrations into NP suspensions, ranging from 0.03 ng/mL to 32 µg/mL was added. PLL, plain NPs and plain NP-PLL were also tested in equivalent concentrations to RIF formulations. Controls containing only biofilms (untreated) or medium without cells (blank) were also tested. The microplate was then incubated with treatments at 37 °C aerobically for 24 h. Wells were washed three times with PBS in order to remove unbound bacteria. Wells were then filled with 120 µL of 0.1% CV and the microplate was incubated for 10 min at room temperature. The excess dye was removed by three washes with PBS and the plate was air-dried. The remaining dye was then solubilized by 100 µL of glacial acetic acid at 33% before reading OD at 590 nm. Adhered biofilm biomass revealed by CV staining corresponded to live and dead bacteria and the matrix, and was assessed as a percentage related to the untreated condition.

Bacterial killing activity of RIF, NP-RIF and NP-RIF-PLL was investigated on *S. aureus* biofilms by MTT (Sigma-Aldrich, France) colorimetric assay. 24 h-old biofilms were prepared and treated as for CV assay. After 24 h of treatment, MTT at 5 mg/mL was added to reach 10% of the culture medium volume and the microplate was incubated 15 min at 37 °C. The resulting formazan crystals were dissolved by 100 µL of a solubilization solution composed of isopropanol (Carlo Erba, France) with 0.25% hydrochloric acid (Sigma-Aldrich, France) and 10% Triton X-100 (Euromedex, France) before reading OD at 570 nm. Biofilm metabolic activity revealed by formazan crystals was correlated to the proportion of live bacteria, and was assessed as a percentage related to the untreated condition.

### NP interaction with planktonic bacteria and antiplanktonic activity

NP interaction with bacteria was evaluated by imaging techniques. Planktonic *S. aureus* was incubated at OD_600_ of 0.008 with red fluorescent NPs without or with PLL coating (NP-Dy650 and NP-Dy650-PLL), at initial solid contents of 0.01 and 0.02%, for 45 min at 37 °C to allow interactions. Bacteria were then centrifuged for 5 min at 1,500 xg and pellets were washed two times with PBS to remove non-interacting NPs. Bacteria were finally stained by Syto9 (Invitrogen, Fisher Scientific, France) at 5 µM for 30 min, and 1% agarose (Euromedex, France) was added to immobilize bacteria on the microplate for their observation by a confocal laser scanning microscopy (CLSM, LSM800, Zeiss) equipped with an AiryScan detector to enhance spatial resolution by 1.7 times in the three dimensions. Excitation lasers were 488 nm for Syto9-labeled bacteria and 640 nm for Dy650-fluorescent NPs. The average red/green ratio of fluorescence intensity was calculated on entire micrographs by an ImageJ (ImageJ software, National Institutes of Health, USA) macro with a subtraction of the background and an automatic detection of areas containing co-localized red and green signals. Fluorescence intensity from bacteria-surrounding NP-Dy650 and NP-Dy650-PLL was related to the one from interacting Syto9-stained bacteria.

To evaluate the antiplanktonic efficacy of rifampicin-loaded NPs without or with PLL coating (NP-RIF and NP-RIF-PLL) on planktonic bacteria after washing, an adapted MIC assay was performed on a 96-well microplate. RIF, NP-RIF or NP-RIF-PLL were diluted at initial RIF concentrations of 4 and 8 µg/mL, corresponding to solid contents of 0.01 and 0.02% for NP suspensions, and mixed volume by volume with bacteria at OD_600_ of 0.008 in BHI. Controls containing only bacteria (untreated) or medium without cells (blank) were also tested. After 45 min of incubation at 37 °C to enable interactions, bacteria were centrifuged for 5 min at 1,500 xg and non-interacting RIF or NPs were washed twice with PBS and fresh BHI was added. The microplate was finally incubated for 24 h at 37 °C and planktonic bacterial growth was assessed by OD_600_ reading as a percentage related to the untreated condition.

### NP diffusion in biofilms

An *S. aureus* suspension was cultured overnight and diluted into BHI supplemented with 1% glucose (Sigma-Aldrich, France) to an OD_600_ of 0.004. One hundred microliters were added in wells of a 96-well tissue culture non-treated microplate and incubated for 24 h at 37 °C under aerobic conditions. Formed biofilms were then washed three times with PBS to remove unbound bacteria, and were stained by Syto9 at 5 µM for 30 min. To evaluate NP diffusion through biofilms, 50 µL of NP-Dy650 and NP-Dy650-PLL with solid contents of 0.25% were carefully deposited by pipetting at the surface of biofilms just prior to CLSM acquisitions (HCS CQ1, Yokogawa). A time-lapse was carried out at 37 °C with sequential acquisitions during 1h30 for layers of biofilm between + 10 and + 28 µm from the bottom of the plate. A similar experiment was realized with only one acquisition after 18 h incubation and bacteria from biofilms were stained by Syto9 5 µM for 30 min before CLSM acquisitions. Excitation lasers were 488 nm for Syto9-labeled bacteria and 640 nm for Dy650-fluorescent NPs. Acquisitions were quantified automatically by an ImageJ macro. A thresholding and a binarization of the green signal from biofilms on entire micrographs enabled to delimit the quantification area of red-NP signal through biofilms. The red fluorescence intensity from NPs was then measured.

### NP accumulation in biofilms and antibiofilm activity

NP accumulation in biofilms was evaluated by imaging techniques. 24 h-old *S. aureus* biofilms were prepared as previously described. Red fluorescent NPs without or with PLL coating (NP-Dy650 and NP-Dy650-PLL), at initial solid contents of 0.0006 and 0.005% were carefully deposited on biofilms by pipetting and incubated for 45 min at 37 °C to allow NP penetration in the deep layers of biofilms and NP interaction with bacteria. Biofilms were then washed two times with PBS to remove non-interacting NPs. Bacteria from biofilms were finally stained by Syto9 at 5 µM for 30 min before CLSM acquisitions (HCS CQ1, Yokogawa). Excitation lasers were 488 nm for Syto9-labeled bacteria and 640 nm for Dy650-fluorescent NPs. Acquisitions were performed for layers of biofilm between + 5 and + 25 µm from the bottom of the plate and were quantified automatically by an ImageJ macro which determines the occupied area and the mean fluorescence intensity from both bacteria and NP signals thanks to an automatic thresholding based on Otsu method by optical section. Overall fluorescence was also measured in another assay replicating the same experimental plan and using a microplate reader (Tecan, Switzerland) by excitation at 486 nm and emission at 506 nm for Syto9-labeled bacteria and by excitation at 660 nm and emission at 674 nm for Dy650-fluorescent NPs. The average red/green ratio of fluorescence intensity was then calculated.

To evaluate the antibiofilm efficacy of rifampicin-loaded NPs without or with PLL coating (NP-RIF and NP-RIF-PLL) on biofilms after washing, an adapted CV assay was performed on a 96-well microplate. 24 h-old *S. aureus* biofilms were prepared as previously described. RIF, NP-RIF or NP-RIF-PLL were diluted at initial RIF concentrations of 0.25 and 2 µg/mL, corresponding to solid contents of 0.0006 and 0.005% for NP suspensions, and 100 µL were poured into wells containing biofilms. Controls containing only biofilms (untreated) or medium without cells (blank) were also tested. After 45 min of incubation at 37 °C to enable attachment, biofilms were washed twice with PBS in order to properly remove non-interacting RIF or NPs, and fresh BHI 1% glucose was added. The microplate was finally incubated for 24 h at 37 °C and adhered biofilm biomass was evaluated by CV staining after washing as a percentage related to the untreated condition.

### Statistical analysis

Experiments were performed in triplicates and repeated two or three times. Data are shown as means ± SD for one representative experiment. Statistical analysis was performed using ANOVA, unpaired and paired *t*-test on GraphPad Prism 6.0 (GraphPad software, USA). A *p-value* < 0.05 was considered as statistically significant.

## Results

### Characterization of drug-loaded and charge-reversed NPs

**Fig. 1:**
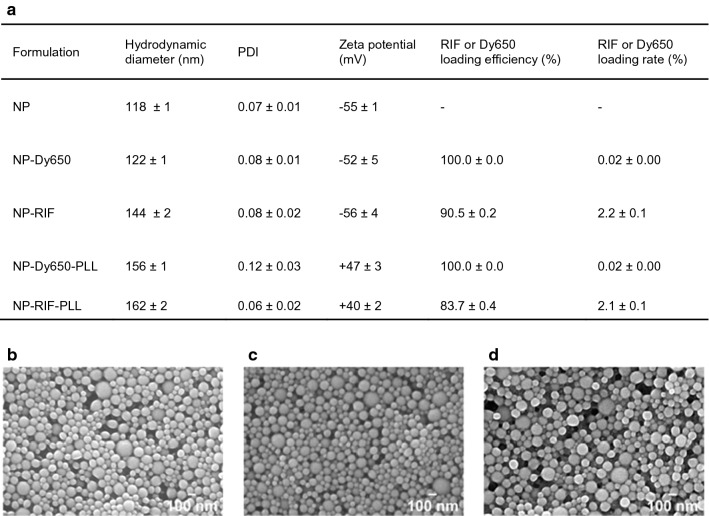
Characterization of NPs functionalized by antibiotic and fluorophore loading and by cationic peptide adsorption. **a** Physicochemical characteristics (hydrodynamic diameter, PDI and zeta potential) and drug loading (loading efficiency and loading rate) of NPs loaded with RIF, Dy650 and PLL. Values are means ± SD of three measurements for one representative experiment out of at least three independent ones. SEM micrographs of NPs (**b**), NP-RIF (**c**) and NP-RIF-PLL (**d**)

Nanoprecipitation was used to produce unloaded and drug-loaded NPs without any surfactant or stabilizer. Plain PLA NPs displayed an average size of 118 nm, as measured by DLS, and a zeta potential of -55 mV, characterizing a highly negative surface charge (Fig. 1a). The low PDI of 0.07 determined by DLS reflected their relatively narrow NP size distribution. The loading of fluorophore (Dy650) or antibiotic (RIF) did not affect PDI and zeta potential values, but slightly increased the size of NPs. Both RIF, at 2.2% loading rate, and Dy650, at 0.02%, were successfully loaded in PLA NPs, resulting in high loading efficiencies of 90 and 100% respectively. When PLL was adsorbed on the surface of NPs, the mean hydrodynamic diameter increased by 10% to 20% to reach 156 and 162 nm for NP-Dy650-PLL and NP-RIF-PLL respectively, without major impact regarding their PDI and their drug loading. Their zeta potential switched from − 52 and − 56 mV for uncoated NPs, to + 47 and + 40 mV, inducing reversal of NP surface charge when formulated with PLL corona. Morphological observations by SEM confirmed the spherical shape of NPs, with no major impact of drug loading or charge reversal on NP morphology and size distribution (Fig. 1b–d).

A cell viability evaluation was performed with NP formulations incubated for 24 h with murine Raw macrophages and NIH/3T3 fibroblasts, using the PrestoBlue reagent. No toxic effect of any plain or formulated NPs was observed for fibroblasts on the range of tested concentrations. All formulations were toxic for phagocytic macrophages, from 0.031 mg/mL for PLL-coated NP regardless of RIF loading, and from 0.125 mg/mL for non-coated NP and NP-RIF (Additional file 1).

### In silico simulation of NP/drug interactions

**Fig. 2:**
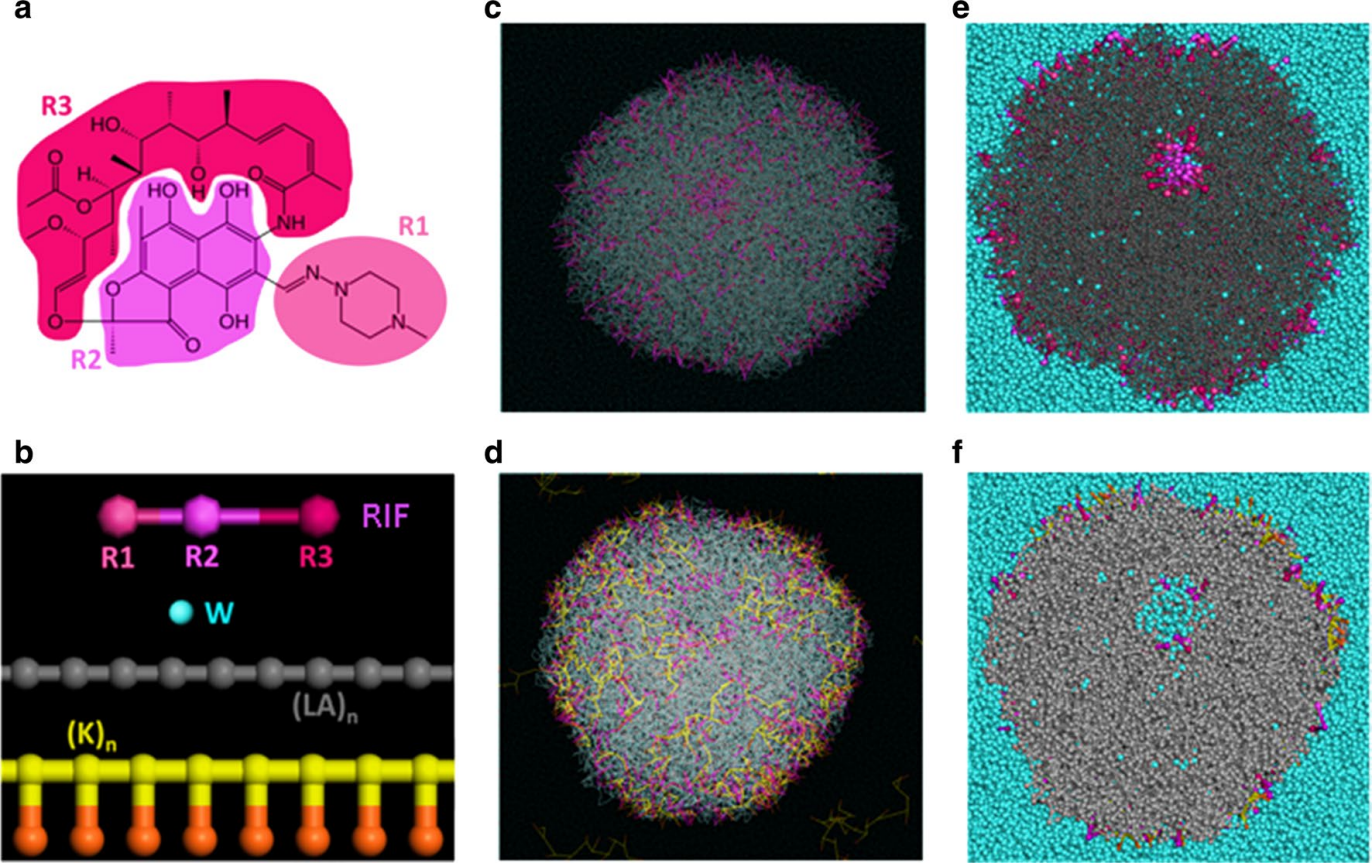
In silico simulation of interactions between rifampicin and PLL within NPs. **a** RIF molecule formula. The groups of atoms corresponding to the R1, R2 and R3 beads are evidenced using colored backgrounds. **b** Schematic representation of the 7 different kind of beads used for the DPD calculations. R1, R2, and R3 for the RIF molecule, water (W) where one bead represents 3 molecules of water, linear chains of lactic acid monomers (LA)_n_ for the PLA chains and linear chains of lysine (K)_n_ for the PLL chains, where the yellow beads represent the backbone of the amino acid chain and the orange beads its side chains. End of the simulation after 30 ns of total simulation time for NP-RIF (**c**) and NP-RIF-PLL (**d**). Section of a newly formed NP-RIF (**e**) and NP-RIF-PLL (**f**)

In silico simulation was performed to further investigate the behavior of RIF and PLL with PLA NPs. DPD simulations allow to model system sizes from nanometers to micrometers and are particularly well adapted to simulate the formulation of compounds within NPs. In order to set up the system and not to create a starting point with an a priori, a 130 Å radius droplet was formed with a mixture of water, PLA chains and RIF molecules, where functional groups were set up as beads (Fig. 2a and b). A layer of water around the droplet was set up in order to avoid interactions between the droplet and its replicative image. Although some small clusters of RIF molecules stayed occasionally trapped with some water molecules inside the newly formed PLA smooth spherical NP, following a random distribution, most of the RIF molecules were localized at the NP surface (Fig. 2c and e). The outer RIF molecules were interacting with the first and second layers of PLA monomers by their R1 and R3 fragments while R2 fragments stayed a little further from the surface. When PLL molecules were added to the water layer surrounding the PLA/RIF/water droplet, most of them ended up located at the surface of the PLA NP with no impact on the RIF behavior (Fig. 2d and f). PLL chains interacted closely with the PLA chains through their backbone fragments, whereas they interacted with the water and some of the RIF molecules through their side chain parts.

The in silico simulation of RIF and PLL distribution provides supportive information for their behavior with NPs and a better understanding of the molecular mechanisms responsible for their encapsulation and adsorption, which appear to be essentially driven through hydrophobic interactions, as previously evidenced [29].

### In vitro drug release from NPs

**Fig. 3:**
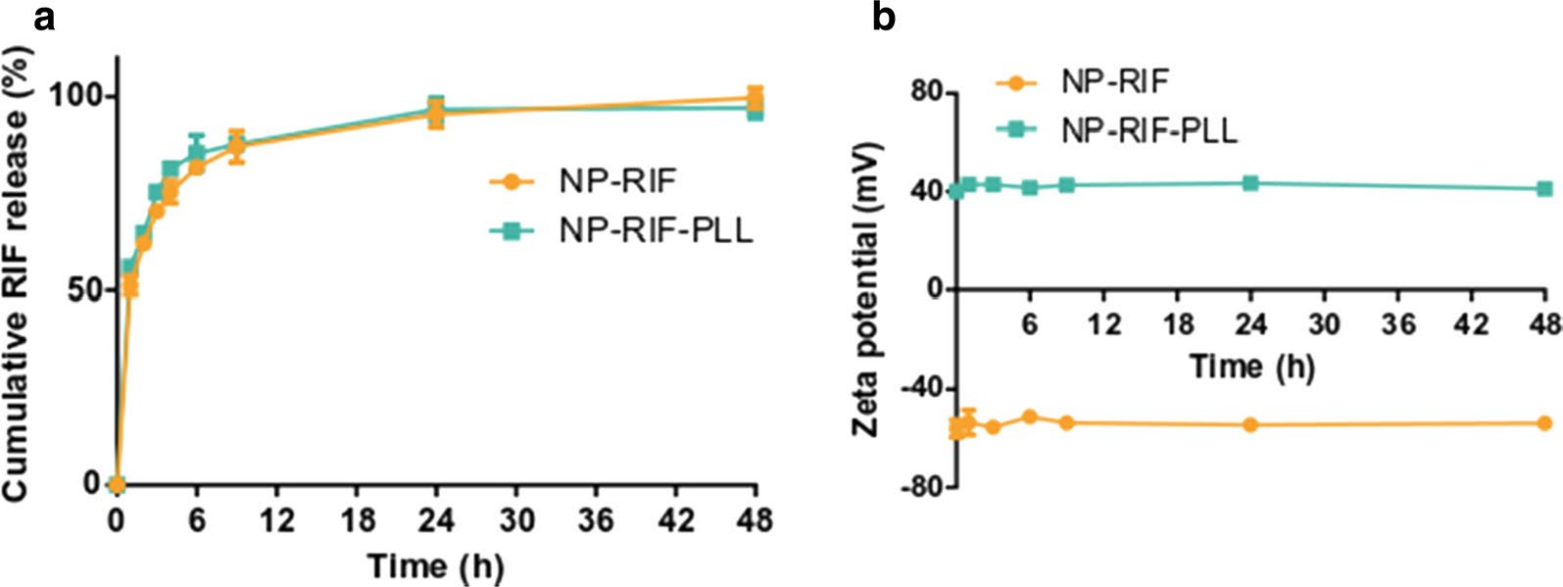
Drug behavior within antibiotic-loaded and PLL-coated NPs in PBS at 37 °C. **a** Cumulative release profile of RIF from NPs and PLL-functionalized NPs in PBS at 37 °C. **b** Zeta potential evolution of charge-reversed and antibiotic-loaded NPs in PBS at 37 °C. Values are means ± SD of three measurements for one representative experiment out of two independent ones

DPD simulations are not really suited to describe long and complex processes such as the release of molecules from NPs. DPD simulations can typically describe molecular evolutions in the timescale of μs or at best ms, such as the encapsulation process, but release is a different process which occurs within hours or days. For this reason, drug release has been exclusively studied through experimental methods. In vitro release of RIF from NPs with or without PLL functionalization was quantified after their dilution in PBS at 37 °C. A similar two-phase kinetic was observed for both formulations with a burst release corresponding to almost 80% of RIF on the 6 first hours. The remaining RIF fully released after 48 h (Fig. 3a). NP surface charge of both formulations, reflected by zeta potential values, was not modified during 48 h in PBS at 37 °C (Fig. 3b). Moreover, in the same conditions, the fluorophore Dy650 did not release from NPs with or without PLL (Additional file 2).

When stored in the bicarbonate buffer at 4 °C, no release of RIF from NPs was observed over the investigated period of 60 days (Additional file 3).

### Antiplanktonic and antibiofilm properties of charge-reversed and antibiotic-loaded NPs

**Fig. 4:**
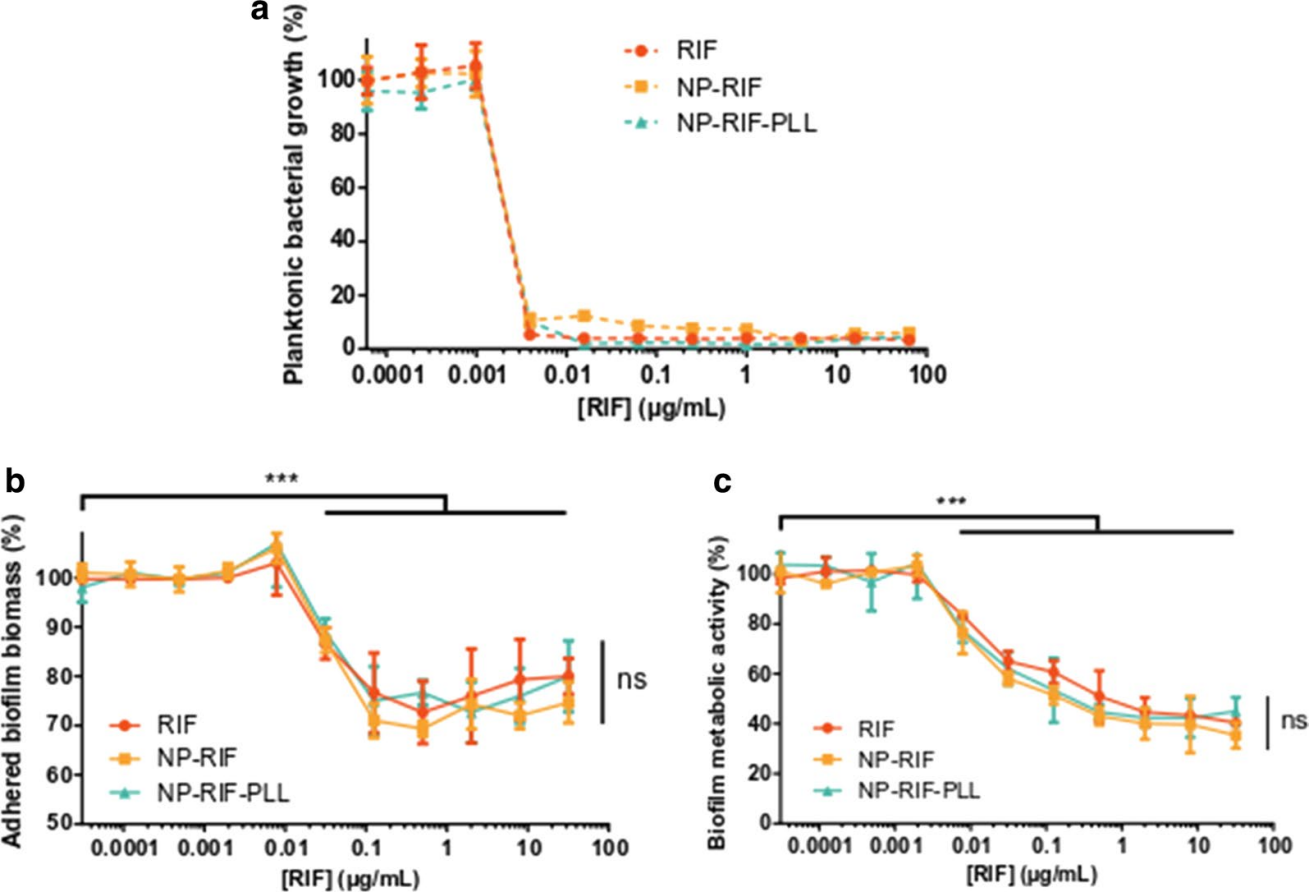
In vitro effect of antibiotic in soluble and formulated forms on *S. aureus* under planktonic and biofilm modes of growth. **a** Planktonic *S. aureus* relative growth after 24 h of co-culture with RIF, NP-RIF or NP-RIF-PLL at different concentrations. **b** Relative adhered biomass of *S. aureus* biofilms stained by CV, after 24 h of co-culture with different concentrations of RIF, NP-RIF or NP-RIF-PLL on 24 h-old biofilms, followed by washing. **c** Relative viability of the bacteria from *S. aureus* biofilms evaluated by MTT, after 24 h of co-culture with different concentrations of RIF, NP-RIF or NP-RIF-PLL on 24 h-old biofilms. Percentages are reported to the untreated condition. Values are means ± SD of three replicates for one representative experiment out of three independent ones. Paired *t*-tests (compared to the untreated condition), *** p < 0.001. ANOVA (between formulations), ns: not significant

To evaluate the antiplanktonic activity of the antibiotic under soluble form or formulated with NPs, its minimum inhibitory concentration (MIC) was evaluated by broth microdilution tests performed on planktonic *S. aureus* with serial dilutions of formulations and free drug. Planktonic bacterial growth was inhibited with a MIC of 0.004 µg/mL for RIF, NP-RIF and NP-RIF-PLL with no statistical significance of RIF encapsulation in NPs or NP-PLL compared to the free antibiotic (Fig. 4a). PLL, plain NPs and plain NP-PLL did not showed any antiplanktonic activity in the range of tested concentrations (Additional file 4a).

Antibiofilm properties of the antibiotic under soluble form or formulated with NPs were evaluated on *S. aureus* biofilms through 1) its impact on biofilm biomass and 2) its killing activity of bacteria under biofilm mode of growth. The capacity of RIF, NP-RIF and NP-RIF-PLL to degrade biofilms was evaluated by CV staining of the whole biofilm biomass after washing of the unbound biomass. Their ability to kill bacteria within the biofilm was evaluated by MTT colorimetric assay, assessing the metabolic activity of bacteria in unwashed biofilms. RIF only induced a partial biofilm degradation since at least 70% of biofilms remained adhered after 24 h of treatment with the highest RIF concentrations compared to non-treated biofilms (Fig. 4b). Moreover, RIF partially killed bacteria from biofilms and 40% of their metabolic activity was still present after 24 h of treatment with the highest RIF concentrations compared to non-treated biofilms (Fig. 4c). Antibiofilm properties of RIF were not impacted by its loading in NPs and by PLL corona on NPs as no statistical difference was observed between groups. PLL, plain NPs and plain NP-PLL did not have any antibiofilm activity in the range of tested concentrations (Additional file 4b and c).

### Increased NP/bacteria interaction by PLL corona enabled to enhance antibiotic delivery to planktonic bacteria

**Fig. 5:**
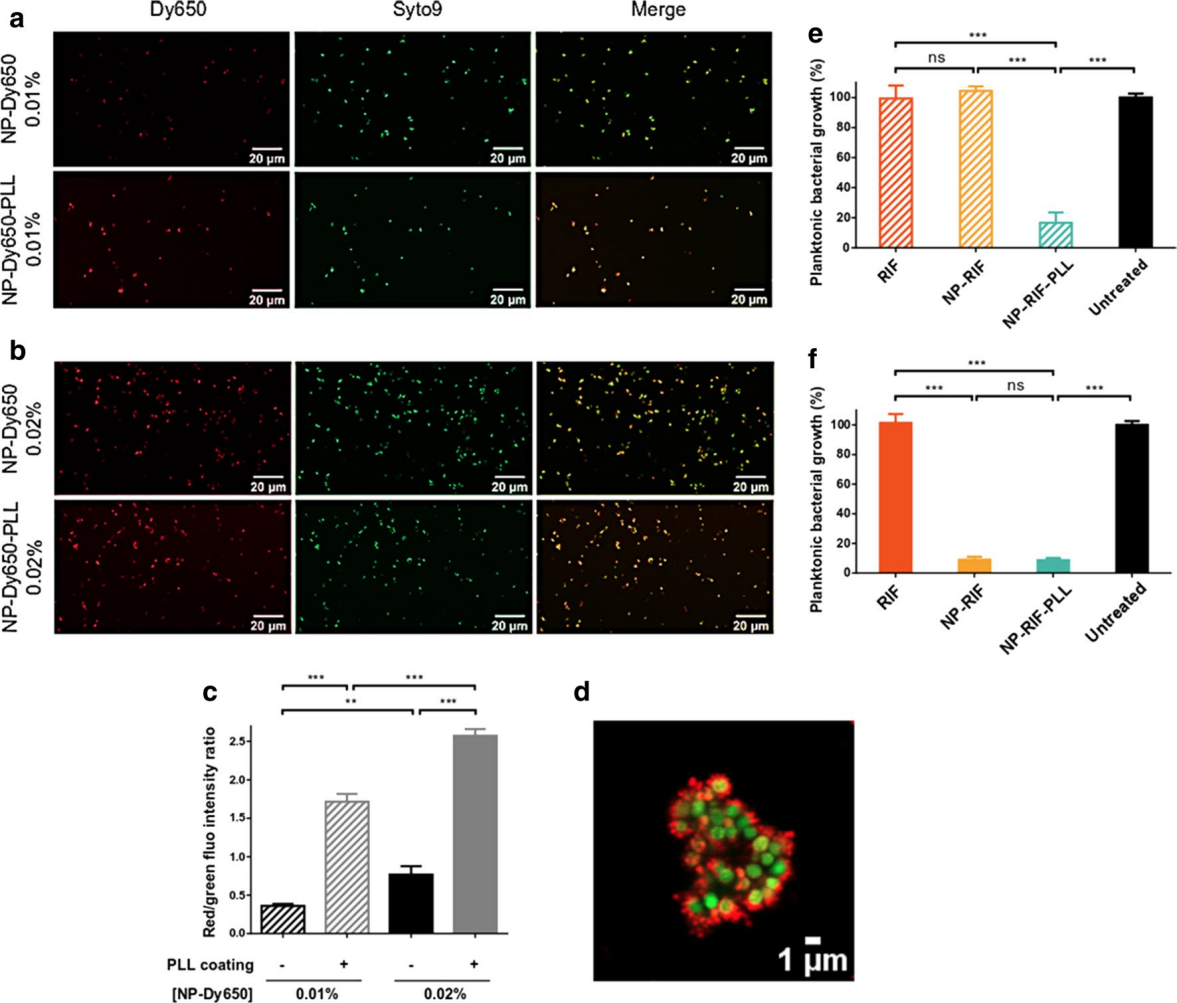
Interaction of NPs and PLL-coated NPs with planktonic *S. aureus*. CLSM micrographs of NP-Dy650 and NP-Dy650-PLL (in red) with initial solid contents of 0.01% (**a**) and 0.02% (**b**) interacting with Syto9-stained bacteria (in green) for 45 min, followed by washing. **c** Ratio of fluorescence intensities from bacteria-surrounding NP-Dy650 and NP-Dy650-PLL related to Syto9-stained bacteria. **d** High resolution micrograph by AiryScan detector on CLSM from **b** of NP-Dy650-PLL (in red) interacting with Syto9-stained *S. aureus* (in green). Relative bacterial growth of planktonic *S. aureus* exposed for 45 min to RIF, NP-RIF or NP-RIF-PLL with initial solid contents of 0.01% (**e**) and 0.02% (**f**), followed by washing to remove poorly-interacting particles and 24 h of incubation. Equivalent RIF concentrations (4 and 8 µg/mL respectively) were used under soluble form. Percentages are reported to the untreated condition. Each picture represents data obtained with three replicates in three independent experiments. Values are means ± SD of three replicates for one representative experiment out of three independent ones. ANOVA, ns: not significant, ** p < 0.01 and *** p < 0.001

The capacity of NPs to interact with planktonic *S. aureus* was investigated by confocal microscopy using red-fluorescent NPs (NP-Dy650 and NP-Dy650-PLL) that were incubated for 45 min with bacteria before washing by selective centrifugations to remove poorly-interacting NPs, followed by green-fluorescent bacteria staining and fixation. The fluorescence intensity from bacteria-surrounding NP-Dy650 and NP-Dy650-PLL was measured and related to the fluorescence intensity from interacting Syto9-stained bacteria. Globally, both NP-Dy650 and NP-Dy650-PLL interacted with bacteria. At low NP/bacteria ratio (Fig. 5a and c), the ratio of red/green fluorescence intensity indicated that NP-Dy650-PLL co-localized almost 4-time more with bacteria than NP-Dy650 that were largely washed away, suggesting more bacterial interactions with PLL-coated NPs. At higher NP/bacteria ratio (Fig. 5b and c), both NP types co-localized more with *S. aureus*, suggesting a dose-dependent interaction between NPs and bacteria. These interactions between red-fluorescent particles and green-fluorescent bacteria were examined in depth by high-resolution imaging (Fig. 5d).

To evaluate the consequences of such interactions on the antiplanktonic efficacy of RIF, an adapted MIC test was carried out. Planktonic *S. aureus* were shortly exposed (45 min) to 4 or 8 µg/mL of RIF in soluble or NP-formulated forms (equivalent to 0.01 and 0.02% of initial solid content), followed by washing and 24 h of incubation at 37 °C in fresh BHI. Planktonic bacterial growth was measured by absorbance at 600 nm. RIF under soluble form did not exert any antiplanktonic activity, as evidenced by levels of planktonic bacterial growth similar to the untreated condition (Fig. 5e and f), and consequently was effectively washed away from bacteria, although doses 1,000 times greater than MIC were used (see results of MIC in the Fig. 4a). At low NP/bacteria ratio (Fig. 5e), NP-RIF were unable to inhibit planktonic bacterial growth, meaning that a significant proportion of NP-RIF was washed away and that the remaining part delivered only sub-inhibitory RIF concentrations to bacteria. On the contrary, NP-RIF-PLL, which have been shown to interact more strongly with bacteria, were able to inhibit planktonic bacterial growth, delivering effective RIF concentrations after a short exposition followed by washing. At higher NP/bacteria ratio (Fig. 5f), non-coated NP-RIF, interacting more with bacteria, inhibited planktonic bacterial growth as well as PLL-coated NP-RIF. The MIC of RIF was reached despite the washing step, suggesting that such concentrations of NP-RIF and PLL-coated NP-RIF allowed sufficient antibiotic-loaded NP retention by interaction with bacteria, to sustainably deliver effective RIF concentrations, after washing.

### Slowed migration of PLL-coated NPs in biofilms

**Fig. 6:**
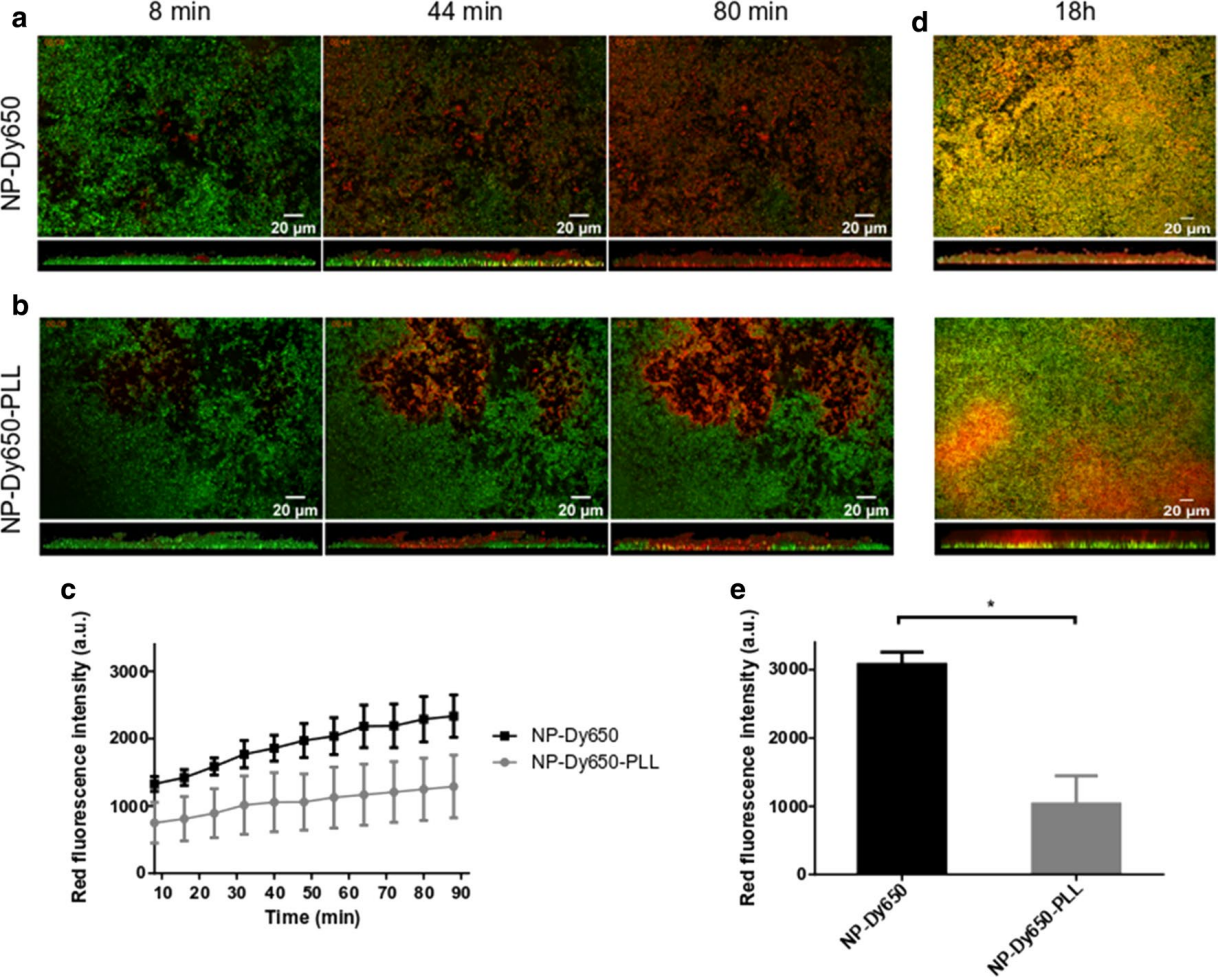
Migration of NPs and PLL-coated NPs in *S. aureus* biofilms. Time-lapse CLSM micrographs of NP-Dy650 (**a**) and NP-Dy650-PLL (**b**) diffusion (in red) into an 18 µm layer thickness (between + 10 and + 28 µm from the plate bottom) of Syto9-stained *S. aureus* biofilm (in green) after 8 (left), 44 (center) and 80 (right) min, with bottom and side views. **c** Kinetic of red-fluorescence NP diffusion into an 18 µm layer thickness of Syto9-stained *S. aureus* biofilm. **d** CLSM micrographs of NP-Dy650 (up) and NP-Dy650-PLL (down) after 18 h diffusion into an 18 µm layer thickness of Syto9-stained *S. aureus* biofilm with bottom and side views. **e** Red fluorescence intensity of NPs into an 18 µm layer thickness of Syto9-stained *S. aureus* biofilm after 18 h diffusion. Each picture represents data obtained with three replicates in three independent experiments. Values on graphs (**c**) and (**e**) are means ± SD of three replicates for one representative experiment out of three independent ones. Unpaired *t*-test, * p < 0.05

The capacity of NPs to migrate into biofilms was evaluated by confocal microscopy through a kinetic evaluation of red-fluorescent NP-Dy650 and NP-Dy650-PLL diffusion into an 18 µm layer thickness of green-stained *S. aureus* biofilms. A time-lapse acquisition to follow NP migration into an 18 µm layer thickness of biofilm was performed, starting 8 min after NP deposition on biofilm surface. NP-Dy650 rapidly, homogeneously and deeply migrated in biofilms within 1h 30 (Fig. 6a and c) while NP-Dy650-PLL stuck at the entry of bacteria-packed clusters in the upper parts of biofilms, inducing a slowest and heterogeneous migration into biofilms (Fig. 6b and c). This less effective dissemination within the biofilm may be due to the cationic surface charge conferred by the PLL peptide adsorbed at the surface of NPs, which may induce strong interactions with the negatively charged wall of bacteria and possibly with anionic extracellular polymeric substances from the biofilm matrix. Other independent acquisitions taken by a single capture 18 h after NP migration into biofilms were carried out to prevent fading of green fluorescence. Within 18 h, while NP-Dy650 fully penetrated the whole biofilms, NP-Dy650-PLL were still blocked in the upper parts of biofilms (Fig. 6d and e).

### Enhanced NP accumulation by PLL corona allowed to improve antibiotic delivery in biofilms

**Fig. 7:**
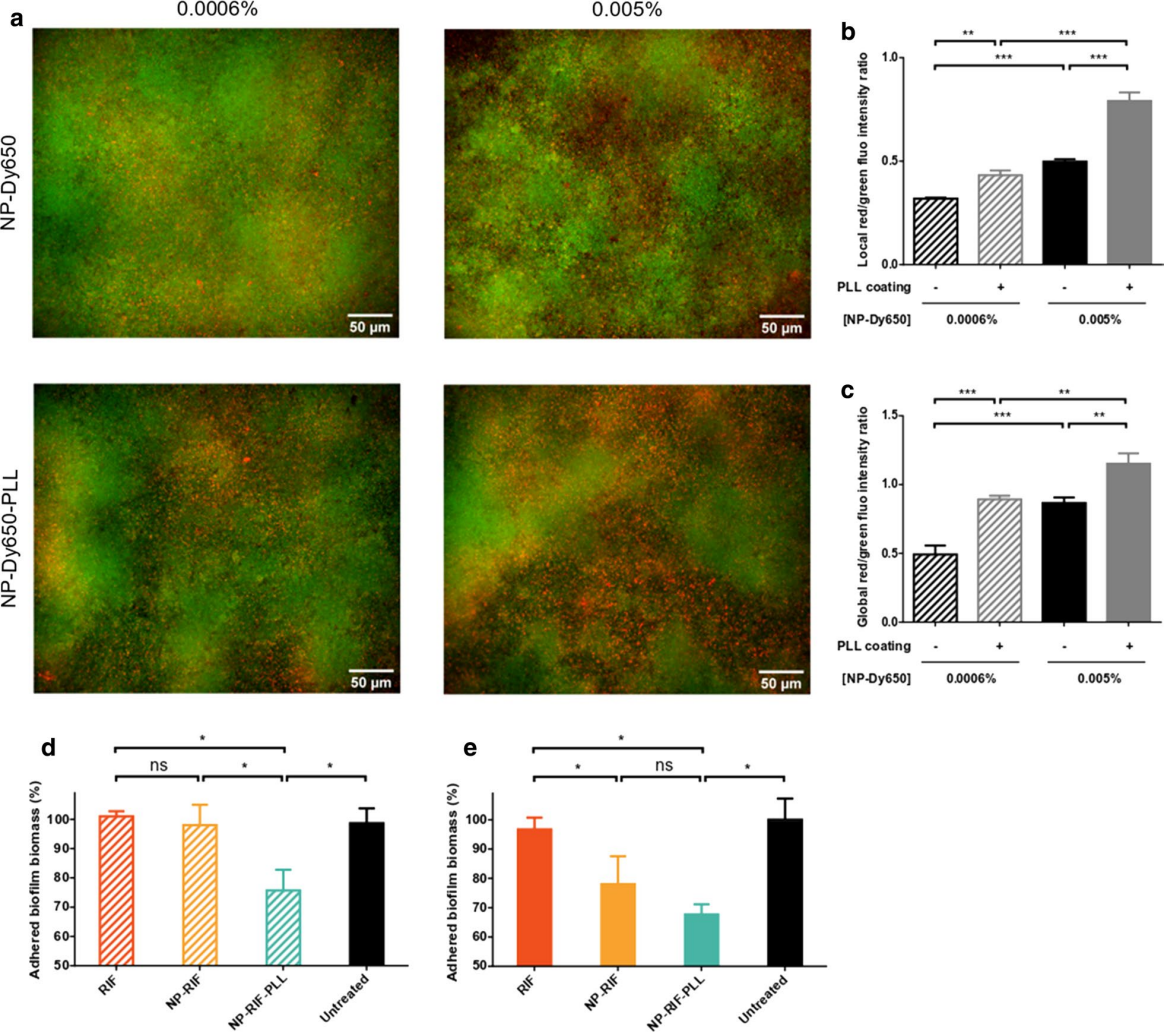
Accumulation of NPs and PLL-coated NPs in *S. aureus* biofilms. **a** CLSM micrographs of NP-Dy650 and NP-Dy650-PLL (in red) with initial solid contents of 0.0006% and 0.005% in a 20 µm layer thickness (between + 5 and + 25 µm from the plate bottom) of Syto9-stained biofilms (in green) and shortly incubated (45 min) before washing. Ratio of fluorescence intensities of remaining NP-Dy650 and NP-Dy650-PLL, related to Syto9-stained biofilms, quantified from CLSM micrographs (**b**) and from measurements by spectrofluorimetry in microplate wells (**c**). Relative adhered biomass of *S. aureus* biofilms stained by CV, after 45 min exposure of 24 h-old biofilms to RIF, NP-RIF or NP-RIF-PLL with initial solid contents of 0.0006% and RIF concentrations of 0.25 µg/mL (**d**) or initial solid contents of 0.005% and 2 µg/mL of RIF (**e**), followed by washing to remove poorly-interacting particles and 24 h incubation. Percentages are reported to the untreated condition. Each picture represents data obtained with three replicates in two independent experiments. Values are means ± SD of three replicates for one representative experiment out of three independent ones. ANOVA, ns: not significant, * p < 0.05, ** p < 0.01 and *** p < 0.001

The highlighted differences in NP/bacteria interaction and in biofilm migration capability of PLL-coated NPs, compared to uncoated NPs, prompt us to assess consequences on their accumulation in biofilms. Thus, we address this hypothesis by introducing a washing step. The capacity of NPs to accumulate in *S. aureus* biofilms was investigated by confocal microscopy using red-fluorescent NPs (NP-Dy650 and NP-Dy650-PLL) that were incubated for 45 min with biofilms to allow their penetration in the deep layers (as assessed in Fig. 6a and 6b), before washing. Bacteria within biofilms were finally stained using the green fluorophore Syto9 before imaging. The fluorescence intensity from remaining NP-Dy650 and NP-Dy650-PLL was quantified on CLSM micrographs and related to the fluorescence intensity from Syto9-stained biofilm bacteria (Fig. 7b). The assay was replicated using the same experimental plan and the overall fluorescence intensity in wells was measured using a microplate fluorometer. The red/green fluorescence ratios have been represented in Fig. 7c. Globally, both NP-Dy650 and NP-Dy650-PLL accumulated in biofilms (Fig. 7a-c). At low solid content, the ratio of red/green fluorescence intensity indicated that NP-Dy650-PLL accumulated almost 1.5-time more in biofilms than NP-Dy650 that were largely washed away, suggesting more interactions between PLL-coated NPs and biofilms. At higher solid content, both NP types were able to accumulate in *S. aureus* biofilms, suggesting a dose-dependent interaction between NPs and biofilms. However, significant higher amounts of cationic NPs remained in biofilms after washing, compared to negatively charged NPs.

We have finally investigated whether the improved antibiotic-loaded NP accumulation had any impact on treatment of biofilms. For this purpose, an adapted CV assay to evaluate biofilm degradation was carried out. *S. aureus* biofilms were exposed for 45 min to 0.25 or 2 µg/mL of RIF in soluble or NP-formulated forms (equivalent to 0.0006 and 0.005% of solid content), followed by washing and 24 h of incubation. Relative adhered biofilm biomass was finally stained by CV after washing of unbound biomass. RIF under soluble form did not exert any antibiofilm activity, as evidenced by equivalent levels of remaining adhered biofilm biomass to the untreated condition (Fig. 7d, e), and consequently was effectively washed away from biofilms, despite the high RIF concentrations used which had demonstrated partial antibiofilm efficacy in the absence of washing step (see results in the Fig. 4b). At low NP solid content (Fig. 7d), NP-RIF were unable to exert any antibiofilm activity. This result means that a significant proportion of NP-RIF was removed by washing, in spite of the high capacity of these uncoated NPs to rapidly penetrate the deep layers of biofilms, as highlighted in the Fig. 6a. On the contrary, PLL-coated NP-RIF, which have been shown to accumulate more strongly in biofilms, were able to maintain effective RIF concentrations in biofilms after washing (Fig. 7d), reducing the biofilm biomass at the maximal capacity of RIF (Fig. 4b). With higher NP solid content, a similar antibiofilm activity was measured using negatively charged PLA-RIF (Fig. 7e). However, a significant increase of the amount of antibiotic-loaded NPs by a factor eight was required.

## Discussion

Faced with the global burden of bacterial infections related to biofilms and clinical failures of current therapeutics, development of novel strategies is an urgent medical need [5]. Reaching effective antibiotic concentrations in biofilms is crucial to prevent recalcitrance phenomena of biofilms [5]. Drug delivery systems have largely been studied the last few decades, presenting appropriate abilities for biofilm control [10]. However, the causal links between NP physicochemical properties and therapeutic efficacy in the control of biofilms have not been fully described, in particular with regard to the impact of the surface charge of these NPs.

In the present work, an antibiotic was loaded in submicronic PLA NPs and their surface charge was reversed by adsorption of a cationic molecule. The nanoprecipitation method used in this study to synthetize NPs combines simplicity, speed and low energy input [30]. Productions result in highly reproducible batches and long-term stable and homogeneous NPs regardless of the loading of antibiotic in NPs and the adsorption of the cationic peptide. Precisely, the use of sodium bicarbonate in the aqueous phase of nanoprecipitation enabled to reach a pH 8.0 superior to the pKa of the amine (7.9) present in the molecule of rifampicin, avoiding NP aggregation caused by electrostatic interactions with the negative surface charge of NPs.

Molecular modelling provides new insights about the mechanisms of interaction of biologically relevant small molecules formulated with PLA NPs [29, 31]. The molecule of rifampicin was constructed using two hydrophobic and one hydrophilic groups with this simplified approach, and a fairly superficial loading of most of the RIF molecules was described with hydrophobic interactions between the hydrophobic groups of the antibiotic and the NPs while the more hydrophilic group was more exposed to the surrounding molecules of water and stayed close to the NP surface. Only a minority of molecules were deeply encapsulated in the core of NP. These results corroborate with RIF release profile from NPs in PBS at 37 °C (Fig. 3), characterized by a fast and massive initial burst release which reflects an encapsulation with a low affinity, contrary to the fluorophore Dy650, a highly hydrophobic molecule, that did not diffuse and demonstrated strong affinity with NPs. Such burst release of the antibiotic allows to rapidly reach concentrations above the MIC, in prevention of recalcitrance phenomena due to biofilm mode of growth and caused by sub-inhibitory antibiotic concentrations [5]. In addition, RIF was not released from NPs in its synthesis buffer at 4 °C, highlighting the possible effect of ionic strength and temperature for interactions between RIF and NPs. The coating of PLL resulted in hydrophobic interactions between the backbone of the amino acid chain and the surface of NPs while PLL did not interact with RIF molecules. Also, positively charged side chains of PLL turned towards the outside of the particle, interacting with the aqueous media, although possible electrostatic interactions with NPs are not directly taken into account in this simplified model. According to the experimental results, it enabled to effectively reverse the surface charge of NPs to highly positive values, not impacting NP stability and RIF release [32].

The loading of RIF in NPs and the PLL coating on these NPs did not impact antiplanktonic and antibiofilm properties of RIF, consistently with total RIF release under 24 h at 37 °C. However, cationic NPs were found to be more cytotoxic than negative ones for phagocytic cells (macrophages) while they were non-toxic for non-phagocytic ones (fibroblasts) in the same range of concentrations. Coolen et al*.* have highlighted NP uptake by cells through clathrin-mediated endocytosis and phagocytosis [33]. Raw macrophages are therefore more likely to accumulate high doses of NPs than non-phagocytic fibroblasts. Legaz et al*.* have already demonstrated a clear relationship between cell viability and cell uptake of NPs [34]. Indeed, high NP accumulation was associated with a significant decrease of intracellular ATP level, an increase of ROS production and perturbation of the cell cycle. We therefore believe that fibroblasts accumulate fewer NPs than macrophages, which explains these differences in sensitivity. Moreover, positively charged NPs are able to induce stronger mitochondrial and lysosomal damages than anionic NPs but charge density and hydrophobicity are equally important factors [35].

The benefit of PLL coating onto RIF-loaded PLA NPs lies in the ability of such cationic corona to favor NP interactions with planktonic *S. aureus* (Fig. 5a–c). Such non-specific targeting has previously been described for positively charged NPs compared to negative ones, with both Gram-positive and Gram-negative bacteria, suggesting electrostatic interactions with the global negative charge of the bacterial wall among its heterogeneously charged surface [23, 36]. While RIF under soluble form was fully washed from bacteria vicinity, the capability of positively charged NPs to strongly interact with planktonic bacteria enabled to sustainably deliver effective RIF concentrations despite a washing step, and using half quantity that negatively charged ones. Similar results were obtained by Hasan et al*.* using polyethylenimine-coated clindamycin-loaded PLGA NPs. At a solid content of 0.05%, positively charged NPs exerted an antiplanktonic activity after 1 h of incubation and washing compared to the free antibiotic and negatively charged NPs, while they were all effective without washing [23].

While the size of NPs influences their ability to diffuse in biofilms, 130 nm of diameter was previously defined as optimal for biofilm penetration compared to bigger NPs [17, 18]. Surface charge of NPs also plays a crucial role for their interaction with biofilms and may be a determinant aspect for the control of bacterial biofilms [6, 15]. Whereas negatively charged PLA NPs rapidly and homogeneously migrated through the whole *S. aureus* biofilms, positively charged NPs bound on the top of the biofilms and at entry of dense bacteria clusters, impeding their penetration deeper into biofilms. This suggests electrostatic interactions between positive NPs and negatively charged biofilm components that are bacteria [21, 22, 37] and macromolecules from the matrix like eDNA [20, 38]. However, the fast and widespread migration of uncoated NP-RIF went at the detriment of being easily washed out, while positively charged NPs were retained in greater quantities in the biofilm (Fig. 7a–c). Similar electrostatic interactions preventing from wash-out were already described, using micelles, silver nanoclusters or dendrons with different surface compositions [21, 22, 37]. However, in each of these studies, no link was established between biofilm accumulation capability and treatment efficacy. After a short treatment time of *S. aureus* biofilms, RIF under soluble form was fully washed away from biofilms and therefore no longer presented any antibiofilm activity. The capability of negatively and positively charged NPs to accumulate in biofilms was correlated with a dose-dependent antibiofilm effect (Fig. 7d, e). By the effect of surface charge, NP-RIF-PLL sustainably delivered inhibitory RIF concentrations with lower NP concentrations, reaching an antibiofilm activity similar to that observed without washing (Fig. 4b). While the bacterial growth under biofilm lifestyle lead to the failure of current therapeutics, antibiotic inhibitory concentrations have to be greatly increased in biofilms for an effective treatment [10]. Contributing to the maintenance of effective doses by passive electrostatic targeting of NPs is therefore fundamental for the treatment of biofilms.

Overall, both anionic and cationic NPs can offer substantial advantages for biofilm eradication. From the one side, anionic NPs are able to rapidly reach deepest bacteria in biofilms and sustainably deliver antibiotic in time, limiting the apparition of recalcitrance phenomena. From the other side, cationic NPs can interact with bacteria and accumulate in biofilms, limiting their wash-out and maintaining antibiotic efficacy. This formulation could be optimized in the future by evaluating different cationic molecules at the NP surface and characterizing the impact of molecule choice, its molecular weight and surface density on the NP ability to interact and accumulate in biofilms. Further animal experimentations represent also the next step to validate the potential of this strategy. Hasan et al*.* demonstrated that cationic PLGA NPs loaded with clindamycin steeply decreased the bacterial burden of mice wounds infected by *S. aureus* compared to anionic NPs [23]. This result was correlated with a better wound healing characterized by a significantly lower wound size after 8 days of topical treatment.

## Conclusion

Charge-reversed and antibiotic-loaded PLA NPs were designed as strategic tool to treat bacterial infections in a biofilm context, considering the recalcitrance of biofilms toward antibiotics. Stable formulations were successfully synthetized by nanoprecipitation and the size of drug delivery system was chosen to facilitate their migration into biofilms. RIF was highly and superficially loaded into NPs, enabling the delivery of effective antibiotic doses with a two-phase release which is appropriate for biofilm-associated treatments. NP coating with a poly-l-lysine corona inverted surface charge of anionic NP-RIF, leading to positively charged NP-RIF which enabled higher interactions with planktonic *S. aureus* and biofilms than negatively charged NPs. While NP penetration within biofilms plays a crucial role in the biofilm control, this study highlighted the importance of the antibiotic-loaded NP ability to target and to be retained in biofilms. The highest interactions observed with positively charged NPs enabled to retain more antibiotic-loaded NPs in biofilms and to sustainably deliver effective RIF concentrations with lower doses. To our knowledge, this is the first time that a correlation has been established between the ability of NPs to be retained in a biofilm and their anti-biofilm therapeutic efficacy. Efforts can be made to improve these systems and especially reducing their toxicity, for example by surface charge switching using a polymer cationic at acidic pH in biofilms, but anionic at physiological pH. Finally, a better knowledge of the mechanisms governing the efficacy of these systems is a fundamental prerequisite to improve antibiotic delivery in biofilms.

## Supplementary Information

**Additional file 1.** Cytotoxicity of plain, RIF-loaded and PLL-coated NPs. Cell viability of murine Raw 264.7 macrophages (**a**) and NIH/3T3 fibroblasts (**b**), evaluated by PrestoBlue assay. Percentages are reported to the untreated condition. Values are means ± SD of three replicates for one representative experiment out of two independent ones.

**Additional file 2.** Cumulative release profile of Dy650 from NPs and PLL-functionalized NPs in PBS at 37 °C. Values are means ± SD of three measurements for one representative experiment out of two independent ones.

**Additional file 3.** Cumulative release profile of RIF from NPs at 4°C. Values are means ± SD of three measurements for one representative experiment out of two independent ones.

**Additional file 4.** In vitro effect of PLL, non-coated and PLL-coated NPs on S. aureus under planktonic and biofilm modes of growth. **a** Planktonic S. aureus growth after incubation with PLL, plain NPs or plain NP-PLL. **b** Adhered biomass of S. aureus biofilms stained by CV, after incubation with PLL, plain NPs or plain NP-PLL and washing. **c** Bacterial viability of S. aureus biofilms evaluated by MTT, after incubation with PLL, plain NPs or plain NP-PLL. Percentages are reported to the untreated conditions. NP, PLL and NP-PLL were used in equivalent concentrations to RIF formulations. Values are means ± SD of three replicates for one representative experiment out of three independent ones.

## Data Availability

All data generated or analyzed during this study are included in this published article and its supplementary information files.

## Abbreviations

BHI: Brain heart infusion
CLSM: Confocal laser scanning microscopy
CV: Crystal violet
DLS: Dynamic light scattering
DPD: Dissipative particle dynamics
MIC: Minimal inhibitory concentration
MW: Molecular weight
NPs: Nanoparticles
NP-Dy650: Dy650-loaded nanoparticles
NP-Dy650-PLL: Dy650-loaded nanoparticles coated with PLL
NP-RIF: RIF-loaded nanoparticles
NP-RIF-PLL: RIF-loaded nanoparticles coated with PLL
OD: Optical density
PBS: Phosphate buffer saline
PDI: Polydispersity index
PLA: Poly(d,l-lactic acid)
PLGA: Poly-lactic-co-glycolic acid
PLL: Poly-l-lysine
RIF: Rifampicin
