# Supplementary material for: Surface charge modulation of rifampicin-loaded PLA nanoparticles to improve antibiotic delivery in Staphylococcus aureus biofilms
Source: J Nanobiotechnology. 2021 Jan 7;19:12. doi: 10.1186/s12951-020-00760-w (PMC7792288; doi:10.1186/s12951-020-00760-w)
Supplement: Supplementary file 1 — Additional file 1. Cytotoxicity of plain, RIF-loaded and PLL-coated NPs. Cell viability of murine Raw 264.7 macrophages (a) and NIH/3T3 fibroblasts (b), evaluated by PrestoBlue assay. Percentages are reported to the untreated condition. Values are means ± SD of three replicates for one representative experiment out of two independent ones. [file 12951_2020_760_MOESM1_ESM.docx]

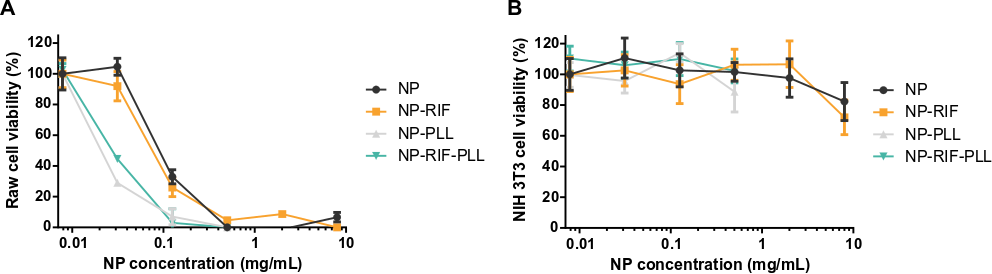


**Additional file 1.** Cytotoxicity of plain, RIF-loaded and PLL-coated NPs. Cell viability of murine Raw 264.7 macrophages (A) and NIH/3T3 fibroblasts (B), evaluated by PrestoBlue assay. Percentages are reported to the untreated condition. Values are means ± SD of three replicates for one representative experiment out of two independent ones.
