# Supplementary material for: Surface charge modulation of rifampicin-loaded PLA nanoparticles to improve antibiotic delivery in Staphylococcus aureus biofilms
Source: J Nanobiotechnology. 2021 Jan 7;19:12. doi: 10.1186/s12951-020-00760-w (PMC7792288; doi:10.1186/s12951-020-00760-w)
Supplement: Supplementary file 2 — Additional file 2. Cumulative release profile of Dy650 from NPs and PLL-functionalized NPs in PBS at 37 °C. Values are means ± SD of three measurements for one representative experiment out of two independent ones. [file 12951_2020_760_MOESM2_ESM.docx]

**Additional file 2.** Cumulative release profile of Dy650 from NPs and PLL-functionalized NPs in PBS at 37°C. Values are means ± SD of three measurements for one representative experiment out of two independent ones.
