# Supplementary material for: Surface charge modulation of rifampicin-loaded PLA nanoparticles to improve antibiotic delivery in Staphylococcus aureus biofilms
Source: J Nanobiotechnology. 2021 Jan 7;19:12. doi: 10.1186/s12951-020-00760-w (PMC7792288; doi:10.1186/s12951-020-00760-w)
Supplement: Supplementary file 3 — Additional file 3. Cumulative release profile of RIF from NPs at 4°C. Values are means ± SD of three measurements for one representative experiment out of two independent ones. [file 12951_2020_760_MOESM3_ESM.docx]

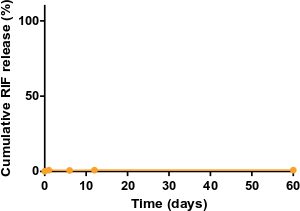


**Additional file 3.** Cumulative release profile of RIF from NPs at 4°C. Values are means ± SD of three measurements for one representative experiment out of two independent ones.
