# Supplementary material for: Surface charge modulation of rifampicin-loaded PLA nanoparticles to improve antibiotic delivery in Staphylococcus aureus biofilms
Source: J Nanobiotechnology. 2021 Jan 7;19:12. doi: 10.1186/s12951-020-00760-w (PMC7792288; doi:10.1186/s12951-020-00760-w)
Supplement: Supplementary file 4 — Additional file 4. In vitro effect of PLL, non-coated and PLL-coated NPs on S. aureus under planktonic and biofilm modes of growth. a Planktonic S. aureus growth after incubation with PLL, plain NPs or plain NP-PLL. b Adhered biomass of S. aureus biofilms stained by CV, after incubation with PLL, plain NPs or plain NP-PLL and washing. c Bacterial viability of S. aureus biofilms evaluated by MTT, after incubation with PLL, plain NPs or plain NP-PLL. Percentages are reported to the untreated conditions. NP, PLL and NP-PLL were used in equivalent concentrations to RIF formulations. Values are means ± SD of three replicates for one representative experiment out of three independent ones. [file 12951_2020_760_MOESM4_ESM.docx]

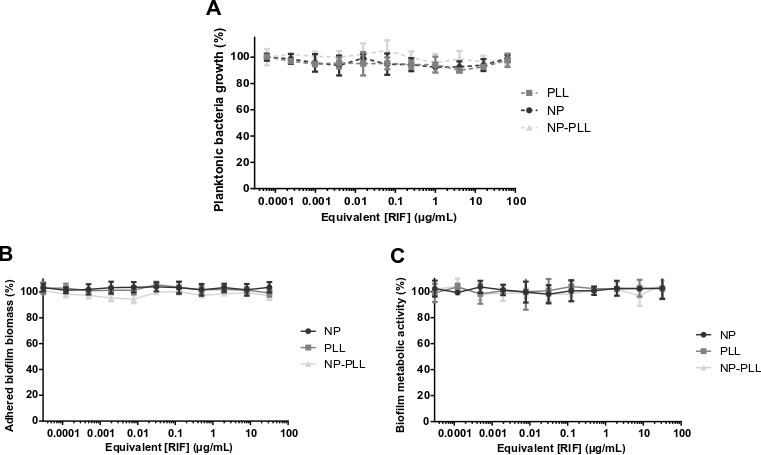


**Additional file 4.** In vitro effect of PLL, non-coated and PLL-coated NPs on S. aureus under planktonic and biofilm modes of growth.

(A) Planktonic S. aureus growth after incubation with PLL, plain NPs or plain NP-PLL. (B) Adhered biomass of S. aureus biofilms stained by CV, after incubation with PLL, plain NPs or plain NP-PLL and washing. (C) Bacterial viability of S. aureus biofilms evaluated by MTT, after incubation with PLL, plain NPs or plain NP-PLL. Percentages are reported to the untreated conditions. NP, PLL and NP-PLL were used in equivalent concentrations to RIF formulations. Values are means ± SD of three replicates for one representative experiment out of three independent ones.
